# A Survey on Green Enablers: A Study on the Energy Efficiency of AI-Based 5G Networks

**DOI:** 10.3390/s24144609

**Published:** 2024-07-16

**Authors:** Zeinab Ezzeddine, Ayman Khalil, Besma Zeddini, Habiba Hafdallah Ouslimani

**Affiliations:** 1 Laboratory of Electrochemistry of Materials for Energetics (LEME) EA 4416, University Paris, 92410 Nanterre, France; zeinab.ali.ezzeddine@gmail.com (Z.E.); houslima@parisnanterre.fr (H.H.O.); 2 SATIE Laboratory CNRS–UMR 8029, CY Tech, CY Cergy Paris University, 95000 Cergy, France; besma.zeddini@cyu.fr; 3 Adnan Kassar School of Business, Lebanese American University, Beirut 1102-2801, Lebanon

**Keywords:** artificial intelligence (AI), Internet of Things (IoT), key performance indicators (KPIs), machine learning (ML), quality of service (QoS), energy efficiency

## Abstract

In today’s world, the significance of reducing energy consumption globally is increasing, making it imperative to prioritize energy efficiency in 5th-generation (5G) networks. However, it is crucial to ensure that these energy-saving measures do not compromise the Key Performance Indicators (KPIs), such as user experience, quality of service (QoS), or other important aspects of the network. Advanced wireless technologies have been integrated into 5G network designs at multiple network layers to address this difficulty. The integration of emerging technology trends, such as machine learning (ML), which is a subset of artificial intelligence (AI), and AI’s rapid improvements have made the integration of these trends into 5G networks a significant topic of research. The primary objective of this survey is to analyze AI’s integration into 5G networks for enhanced energy efficiency. By exploring this intersection between AI and 5G, we aim to identify potential strategies and techniques for optimizing energy consumption while maintaining the desired network performance and user experience.

## 1. Introduction

5G is the fifth generation of wireless communication technology, following 4G. In contrast to 4G, 5G is designed to support a diverse range of applications, including massive Internet of Things (IoT) deployments, ultra-reliable low-latency communications, and high-bandwidth multimedia services. Energy efficiency is a critical consideration in modern networks, including 5G. As the demand for data and connectivity continues to grow exponentially, reducing energy consumption has grown in significance [1].

The design of 5G networks contributes to energy consumption in various ways. For example, 5G utilizes new radio technologies, such as massive Multiple Input, Multiple Output (MIMO); beamforming; and advanced modulation schemes, which enable more efficient use of available spectrum and transmit power [2]. Green enablers are integrated into the architecture of 5G networks to enhance energy efficiency. These techniques help improve spectral efficiency and overall network capacity, leading to reduced energy per bit transmitted. Energy-efficient 5G networks can help minimize the environmental impact, reduce operational costs, and support sustainable development.

In addition to the network design and green enablers, emerging technologies like AI and IoT play significant roles in enhancing energy efficiency in 5G networks. Employing AI algorithms can optimize resource allocation, power control, and network management, resulting in more intelligent and energy-efficient operations [3]. The landscapes of AI and ML can be seen as potential drivers in the automation and optimization of network performance and management complexity [4]. 

At the core of the complementary connection between AI and 5G is the significance of data; 5G opens a floodgate for data, something AI could then analyze as well as learn from more swiftly to create unique consumer experiences that are already suited to the numerous needs of consumers [5]. In the assessment of techniques used to make the 5G network more energy-efficient while enhancing KPIs, the role of AI becomes crucial. AI can optimize network operation and maintenance. For example, network automation techniques include a self-organizing network (SON), which uses AI to predict network behavior and control the network based on these findings [6]. 

Through AI-driven SON, the 5G infrastructure can continuously monitor performance metrics and automatically adjust network parameters to achieve the desired KPIs. AI algorithms can detect anomalies, identify bottlenecks, and implement corrective actions in real time, which leads to efficient network operation with reduced energy overheads.

In this survey, the objective is to conduct an in-depth analysis of the existing AI technologies employed in various 5G enablers and their impact on energy consumption. AI technologies may include ML algorithms, deep learning (DL) models, natural language processing, computer vision, and other AI techniques that are applied in the context of 5G enablers. This investigation will also consider the different components and applications of 5G, such as IoT and virtual reality. The energy consumption analysis will involve assessing the power requirements of the AI technologies integrated into 5G enablers. Potential challenges and limitations in implementing AI technologies in 5G enablers will be highlighted.

The paper makes significant contributions in several aspects:

- Detailed discussion of AI technologies and green enablers: This paper provides a comprehensive analysis of AI technologies and green enablers across various network layers. It explores their roles and capabilities in enhancing energy efficiency, offering a detailed roadmap for future researchers in the field.
- Impact on energy consumption: It discusses the profound impact of these technologies on reducing energy consumption in network operations. By highlighting their importance, this paper underscores their potential to revolutionize energy efficiency practices.
- Summary of implemented methods: This paper summarizes various methods implemented to achieve energy efficiency in networks. It evaluates their effectiveness and contribution to reducing energy consumption, providing valuable insights into best practices.
- Future directions: Finally, this paper outlines future research directions in the domain of AI technologies and green enablers. It identifies potential areas for improvement and innovation, guiding researchers towards new opportunities and challenges.

In summary, this survey aims to contribute to the understanding of the relationship between AI technologies and energy consumption in the context of 5G enablers, paving the way for more sustainable and efficient 5G networks in the future. 

**Figure 1:**
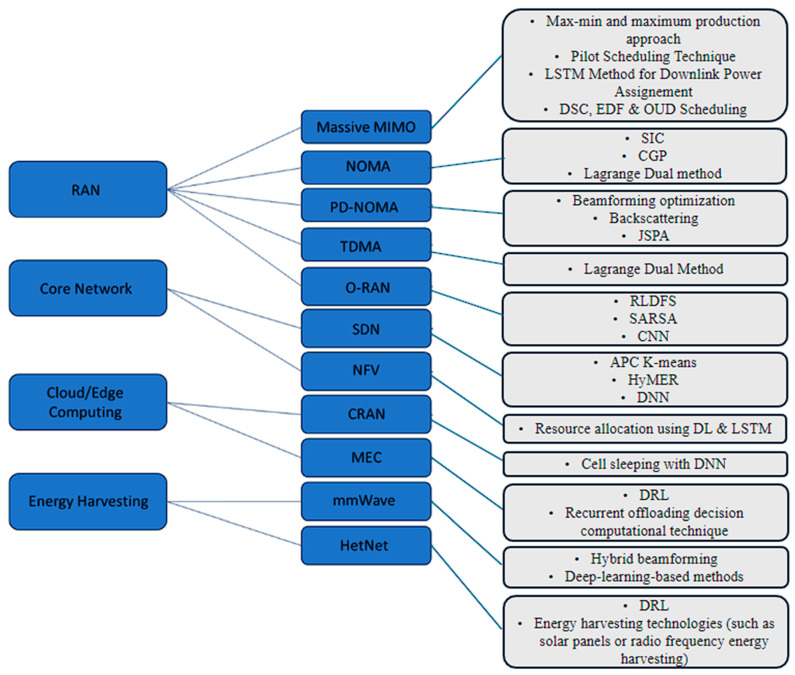
AI techniques flow chart.

Figure 1 shows a flow chart mentioning the AI techniques currently used in each 5G enabler in different 5G network layers, which will be discussed in detail through this survey.

## 2. Paper Outline

This paper is organized into three main sections (Section 3, Section 4 and Section 5). In Section 3, we delve into the different layers of the 5G network, discussing the specific enablers utilized in each layer and their distinctive characteristics. The categorization of the network layers and AI techniques systematically addresses the implementation of energy-efficient technologies within 5G networks. Additionally, we highlight the utilization of AI techniques to improve energy efficiency and enhance KPIs across the network. We underscore the significance of energy efficiency in the implementation of environmentally friendly techniques within the 5G network. 

Each part includes a description of the technology and an analysis of the related work. In Section 4, an overview of potential directions and challenges that can guide future research in this field is presented. This section aims to assist scholars in their ongoing exploration of the subject matter, offering insights into areas that require further investigation and highlighting obstacles that need to be overcome. Finally, Section 5 concludes the paper by summarizing the key findings and contributions of this research. By structuring the paper in this manner, we provide a comprehensive exploration of the 5G network, from its fundamental layers and enablers to the application of AI techniques for energy efficiency enhancement, while also guiding future research endeavors.

## 3. Fifth-Generation Network Layers and Green Enabler Technologies

The term “5G green enablers” refers to the various technologies, strategies, and practices that contribute to making 5G networks more environmentally friendly and energy efficient. These enablers aim to minimize the carbon footprint and energy consumption associated with 5G network deployments. Fifth-generation green enablers can be implemented across various layers of the fifth-generation network to achieve energy efficiency and environmental sustainability. Table 1 shows a summary of the main ML techniques in various 5G enablers that will be discussed throughout this paper.

### 3.1. Radio Access Network (RAN)

Information and Communication Technology (ICT) was responsible for 2% to 10% of the world’s energy consumption in 2007, and it is expected to continue to grow. Also, more than 80% of ICT is from RAN, which is deployed to meet the peak traffic load and stay on it even when the load is light. Motivated by saving energy for green communication, 5G specifications require that energy use decreases to 10% that of traditional 4G/Long-Term Evolution (LTE) networks. This objective can be achieved by reducing the power consumption of the base stations and mobile devices [7]. In the RAN layer of 5G, several technologies are used to enhance network performance, capacity, and energy efficiency. Dynamic resource allocation optimizes resource usage across RANs by leveraging AI to analyze and predict network traffic data using techniques like Linear Regression and the Autoregressive Integrated Moving Average [8]. This enables optimal resource allocation, reducing energy consumption. Smart sleep modes intelligently put access points or base stations to sleep based on traffic load patterns, further conserving energy during low-demand periods [8]. While not all these technologies are explicitly considered “green enablers”, they contribute to improving energy efficiency and sustainability in various ways:

- Energy-efficient hardware: Energy-efficient base station (BS) equipment, such as power amplifiers and antennas, can be deployed to reduce power consumption.
- Dynamic power management: Intelligent power control algorithms can be implemented to adjust the transmission power levels of BSs and small cells based on real-time network conditions and user demands. In [9], optimizing WLAN energy consumption was addressed through the dynamic control of access stations, including switching them on/off and adjusting their power based on realistic traffic patterns. The study proposes several Integer Linear Programming (ILP) optimization models and heuristic algorithms for selecting optimal network configurations to minimize energy usage. While ILP models yield better instantaneous power consumption and monthly energy savings, heuristic algorithms significantly reduce computational time and provide feasible solutions quickly, making them suitable for real-world network management [9]. Both approaches ensure sufficient coverage and capacity for active users. Numerical results indicate that the heuristic approach is slightly less effective in energy savings but offers a practical alternative due to its faster computational time [10].
- Virtualization and cloud computing: Network function virtualization (NFV) and Software-defined networking (SDN) can be utilized to virtualize and centralize certain network functions, optimizing resource utilization and reducing energy consumption.
- AI Optimization: AI techniques can be employed to optimize radio resource management, including intelligent scheduling and power allocation algorithms that minimize energy consumption while ensuring QoS.
- Usage of renewable energy sources: A wireless sensor network for the remote monitoring of green Base Station Systems (BSSs) powered by renewable energy source (RES) systems was proposed in [11]. This system provides real-time and historical data, enabling detailed performance analyses and highlighting the need for careful capacity planning to prevent power outages. Linear models show significant CO_2_ reductions from switching to RES-powered BSSs. Additionally, a novel approach for reducing fuel consumption through regulated generator activity and a free-cooling system is proposed, demonstrating further cost and emissions savings [11].

The implementation of each green enabler in the RAN layer will be discussed, followed by how they contribute to energy efficiency.

#### 3.1.1. Massive MIMO

**Figure 2:**
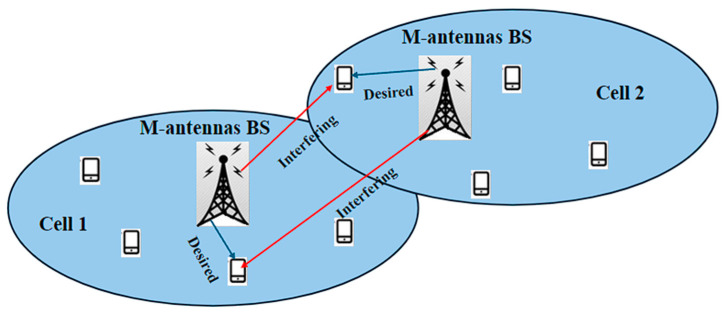
Illustration of massive MIMO.

Massive MIMO utilizes a large number of antennas at the BS to improve spectral efficiency, increase network capacity, enhance coverage, and improve data rate, throughput, and energy efficiency while lowering latency on future mobile devices. It enables beamforming techniques, directing wireless signals towards specific users, thereby reducing interference and improving energy efficiency. It directs wireless energy to specific users using tight beams, as opposed to typical MIMO systems that employ large beam sectors, which require the use of Higher-Power Amplifiers (HPAs) and absorb most of the BS’s energy [1]. However, the problem of interference persists in massive MIMO when hundreds of channels exist at one BS. Figure 2 illustrates the interference that occurs in massive MIMO. Moreover, there is further spatial complexity [3]. 

Fifth-generation networks can concentrate the transmitting and receiving of signal energy in small areas of space by utilizing a considerable number of antennas. ML and DL have been investigated for optimizing the weights of antenna elements in massive MIMO. They can predict the user distribution and accordingly optimize the weights of antenna elements, which can improve the coverage in a multi-cell scenario [7]. The use of a reasonable number of pilots and simple estimation methods for accurate channel estimation is difficult in massive MIMO. The DL technique for channel estimation could be utilized to map channels in frequency and space, as the authors indicated in [12]. Therefore, to save bandwidth, the same pilot patterns are regularly provided to users in distinct cells for short-term coherence, but this creates the problem of pilot contamination. Pilot pollution has become one of the leading causes of performance loss in massive MIMO, leading to dropped calls and mobility issues, which are considered important KPIs. 

In [13], a deep learning-based approach allows the network to allocate downlink power based on the User Equipment (UE) location. Several power-allocation strategies were inefficient, including max–min and maximum production, which were remedied by using a different neural network, the Long Short-Term Memory (LSTM) layer [3]. Despite the promising results of the simulation in terms of power allocation, the weakest point in massive MIMO efficiency remains in the real-time environment. A pilot scheduling technique used in massive MIMO systems led to a reduction in pilot contamination. Users experiencing channel defects face communication interruptions; therefore, a pilot scheduling scheme is proposed that combines user grouping according to different levels of pilot contamination. Currently, most studies concern the spectrum efficiency of hybrid precoding, which reduces the radio frequency (RF) chains’ huge energy consumption in the massive MIMO system. 

Incorporating massive MIMO into the RAN and employing the DL method LSTM to allow the system to assign downlink power based on the location of the user’s broadband access in 5G KPIs. The technology is expected to have 1000 times the bandwidth of current LTE and LTE-Advanced, ultra-low latency of 1 millisecond, 90% energy savings, 10 times longer battery life, and 10 to 100 times greater peak user data speeds, all with cost-effective equipment [14]. 

Combined with scheduling techniques, beamforming enhances multi-user performance by directing the signal towards specific users, optimizing resource utilization. Packet scheduling plays a crucial role in the performance of smart grid communications by managing bandwidth resource allocation [15]. The combination of scheduling and beamforming techniques can enhance multi-user performance in communication systems.

In [15], the authors evaluate the Deadline Scheduling with Commitment (DSC) approach and demonstrate that the popular Earliest Deadline First (EDF) scheduling technique can be significantly improved. A novel Optimal Usage and Dropping (OUD) scheduling approach is proposed for efficient resource block (RB) utilization, meeting the stringent requirements of smart grid communications. Performance metrics indicate that the OUD approach outperforms both the improved EDF and DSC techniques. Simulation results show that while the DSC technique has poorer schedule ability compared to the improved EDF, the OUD technique delivers superior performance. The OUD approach is expected to enhance real-time guarantees in smart grid communication systems, allowing them to accommodate more tasks. Future modifications using artificial intelligence tools like Genetic Algorithms could make the OUD algorithm more general, flexible, and intelligent, further optimizing its performance [15].

The following are ways to enhance energy efficiency in MIMO:

- Resource optimization: By accurately predicting channel conditions, traffic patterns, and interference levels, LSTM models can enable more efficient allocation of radio resources in the MIMO system. This optimized resource allocation reduces unnecessary transmissions, leading to lower energy consumption.
- Power control: LSTM-based predictions of traffic demands can be made, and user behavior can inform dynamic power control strategies. By adjusting transmit power levels based on predicted traffic loads and channel conditions, unnecessary power consumption can be avoided. This adaptive power control helps in achieving energy savings without compromising the QoS.

#### 3.1.2. Non-Orthogonal Multiple Access (NOMA)

**Figure 3:**
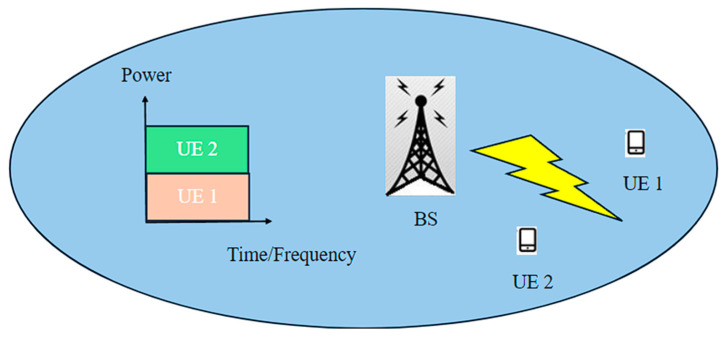
Description of NOMA architecture.

NOMA is a key technology in 5G networks that enhances spectral efficiency and capacity by allowing multiple users to share the same time–frequency resources, as shown in Figure 3. The orthogonality property, which indicates that multiple users do not interfere when accessing network resources, is common in several multiplexing methods [16]. Unlike traditional Orthogonal Multiple Access (OMA) schemes like Frequency Division Multiple Access (FDMA) for the first-generation systems, Time Division Multiple Access (TDMA) for the second generation, and Code Division Multiple Access (CDMA) for the third generation, NOMA enables simultaneous transmissions in the same resource block [17]. 

The adoption of NOMA in 5G networks provides several benefits, including increased system capacity, reduced latency, and enhanced user experience. It enables more efficient utilization of available resources and supports a larger number of connected devices. Implementing NOMA in practical 5G networks involves several challenges. These include the design of efficient power allocation algorithms, interference management, and the need for advanced receiver techniques such as Successive Interference Cancelation (SIC). Additionally, NOMA requires accurate channel state information for power allocation and user scheduling.

The energy optimization strategy in [18] relies on deploying NOMA in RAN while using Complementary Geometric Programming (CGP). The simulation findings show that NOMA is more power-efficient than Orthogonal FDMA (O-FDMA). NOMA improves power efficiency by up to 45–54 percent. This demonstrates the usefulness of NOMA in obtaining improved energy efficiency while keeping the Virtualized Wireless Networks (VWN) slices’ isolated. The proposed method beats O-FDMA in terms of necessary transmit power, particularly when most users are located near the cell edge and channel conditions vary widely. SIC can help some users at the sub-carrier level by reducing interference. However, the minimum needed throughput of each user behaves as a constraint. The other major difficulty in a VWN, which aims to maximize spectrum and infrastructure efficiency, is to keep users in different slices isolated.

Adopting the Lagrange dual method joint time and power allocation in RAN showed that TDMA beat NOMA, demonstrating that TDMA is more spectrum- and energy-efficient. NOMA requires longer (or equal) downlink time than TDMA, consumes more (or equal) energy, and has lower spectral efficiency, as examined in [19].

#### 3.1.3. Power Domain NOMA

Power domain NOMA (PD-NOMA) is a key technology in 5G networks that enables multiple users to simultaneously access the same time-frequency resource. NOMA allocates different power levels to different users within the same resource block, allowing for improved spectral efficiency and increased capacity compared to traditional Orthogonal Multiple Access schemes.

In PD-NOMA, users with stronger channel conditions are allocated higher power levels, while users with weaker channel conditions are allocated lower power levels. This enables multiple users to share the same resource block by exploiting the power domain for separation. The receiver then uses advanced multi-user detection techniques to decode the signals from different users.

To enhance energy efficiency in PD-NOMA, ML techniques can be employed to allow channel prediction, resource allocation, user grouping, power control, and beamforming optimization. 

**Figure 4:**
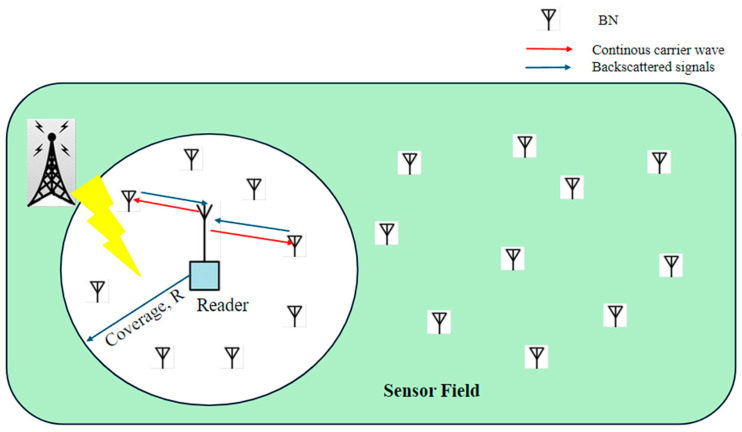
Illustration sensor field of multiple BNs and a reader.

Traditional PD-NOMA systems allow multiplexed subscribers to satisfy the requirements of NOMA at different power levels by using active battery resources [20]. On the contrary, sensor nodes are passive devices with no capability to adjust their transmit power level based on their respective channel gains. In [20], the authors consider a sensor field of multiple Backscatter Nodes (BNs) and a reader, as illustrated in Figure 4. A sensor field of K IoT sensors, BNs, and a reader is considered to adopt a monostatic backscatter communication model to improve network energy efficiency. The contribution of this study is to employ backscatter communication in the field of IoT sensors and to enhance the spectrum efficiency of the system using a hybrid TDMA-based PD-NOMA scheme. Through NOMA, the network increases its spectrum efficiency by multiplexing its nodes in half the time. However, the study succeeded in highlighting important KPIs but failed to emphasize dropped calls, latency, system bandwidth, and mobility. 

An efficient heuristic Joint subcarrier and power allocation scheme (JSPA) was designed by combining the solutions of System Communication Units (SCUSs) and Multiple Channels Per Carrier (MCPC) [21]. In multi-carrier NOMA with cellular power constraints, a novel approach was proposed to overcome the Weak State Routing (WSR) maximization problem. It achieves a near-optimal sum rate with user fairness as well as significant performance improvements compared with NOMA.

In TDMA, the available frequency band is divided into time slots, and each user is allocated exclusive time slots for transmitting and receiving data [22]. This technique has a drawback whereby the use of clock synchronization is essential so that each user can transmit and recuperate their received data without interfering with the other subscribers. This leads to an increase in transmission delays.

#### 3.1.4. Open Radio Access Network (O-RAN)

O-RAN is an emerging architecture and approach in the context of 5G networks. It aims to disaggregate and open traditionally proprietary and integrated network elements, such as the RAN, by introducing open interfaces and standardized hardware and software components. O-RAN is designed to promote interoperability, flexibility, innovation, and cost-effectiveness in the deployment and operation of 5G networks. It breaks down the traditional RAN into functional components, separating hardware and software elements. This allows for the use of standard hardware, such as commercial off-the-shelf servers, and enables the software-defined functionality of the RAN. With the disaggregated architecture, operators can deploy RAN components from different vendors, customize configurations, and optimize network resources based on their unique needs. This flexibility facilitates network optimization and capacity expansion.

Open virtual RAN (open VRAN) refers to a heterogeneous approach to deploying virtualized mobile networks that use open and interoperable protocols and interfaces. It is applied over a common proposed hardware in a multi-vendor software environment, giving greater flexibility than traditional RAN designs. The term “open VRAN” refers to a decentralized strategy for creating virtualized mobile networks. Using open and compatible protocols and interfaces that would be implemented over commonly proposed hardware in a multi-vendor software application drives innovation over traditional RAN designs. The fundamental difficulty in ORAN currently is that the ecosystem is fragmented, ununified, and lacking a clear goal. This issue will persist until there is a convergence of RAN virtualization standards and wireless technology standards (i.e., 3GPP and IEEE) [23]. 

While O-RAN itself is a framework that defines the architecture and interfaces, ML techniques such as the following can be employed within the O-RAN ecosystem to enhance energy efficiency:

- Energy-aware resource allocation: ML algorithms can be utilized to optimize the allocation of network resources, such as power and bandwidth, based on the traffic load, user demand, and energy efficiency objectives. By learning from historical data and network conditions, ML models can dynamically allocate resources to minimize energy consumption while maintaining the desired QoS levels.
- Deep learning: Deep learning refers to the use of deep neural networks (DNNs) with multiple layers to learn complex representations from data. In O-RAN, deep learning techniques, such as convolutional neural networks (CNNs) or recurrent neural networks (RNNs), can be applied for tasks like signal processing, channel prediction, beamforming optimization, and network optimization.

In addition to ML techniques, several AI-based techniques are used in O-RAN to optimize network performance and enhance efficiency, such as reinforcement learning (RL). RL is a subfield of ML where an agent learns through interactions with an environment and receives feedback in the form of rewards or penalties. In O-RAN, RL can be used to optimize dynamic network configuration, power management, and resource allocation based on the received reward signals.

**Figure 5:**
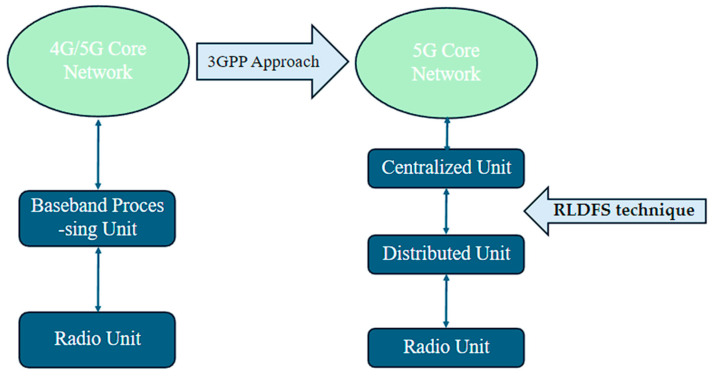
O-RAN architecture with RLDFS technique.

In [24], the authors integrate the O-RAN into the RAN and utilize a reinforcement learning-based dynamic function splitting (RLDFS). With Quality Learning and State–Action–Reward–State–Action (SARSA) algorithms, the RLDFS technique decides which functions to be split between the centralized unit (CU) and the distributed unit (DU) in an O-RAN to make the best use of the RES supply while lowering operating costs. Different solar panels and battery sizes are considered for the network model to evaluate the feasibility of using renewable energy for a Mobile Network Operator (MNO) [23]. This study focused on optimizing energy use by moving renewable energy based on traffic demand and electricity rates. Figure 5 shows the difference between traditional RAN and O-RAN that utilizes the RLDFS technique.

Reinforced Learning-based systems are less expensive than other methods, and their efficacy improves as solar panels and batteries become bigger. The key explanation for this result is the adaptation to a larger proportion of renewable energy. For MNO, the constraint is the battery size. Two methodologies were compared to assess RL algorithms. The first, known as Distributed RAN (D-RAN), uses DUs to handle both Ultra-Reliable Low Latency Communication (URLLC) and Enhanced Mobile Broadband (eMBB) packets. The second, Centralized RAN (C-RAN), handles URLLC packets at DUs to comply with delay requirements and transfers eMBB packets to the CU to save money. However, there was no focus on latency; instead, the authors looked at the impact of solar radiation and battery size on cost [24]. 

QoS is certainly central to decision-making in dynamic function splitting, which concentrates on area traffic capacity. QoS was studied in four main cities with distributed solar radiation in the presence of varied levels of traffic [24]. As a result, it was found that the Q-Learning and SARSA algorithm approaches mentioned above function better when solar radiation rates increase than C-RAN and D-RAN. However, the battery life of UE was not cited. On the other hand, the system bandwidth, spectral data, and peak data rate are important factors that were not explored. Throughput, mobility, and dropped call were also not investigated.

### 3.2. Core Network

In the core network layer of 5G, several technologies are utilized to facilitate efficient network operations and support the connectivity and services provided by the network. While not all of these technologies are inherently green enablers, they can contribute to energy efficiency and sustainability in different ways. Networks protected by SDN and NFV have added a new level of flexibility that allows network operators to provide services with very high requirements across several industries [25].

**Figure 6:**
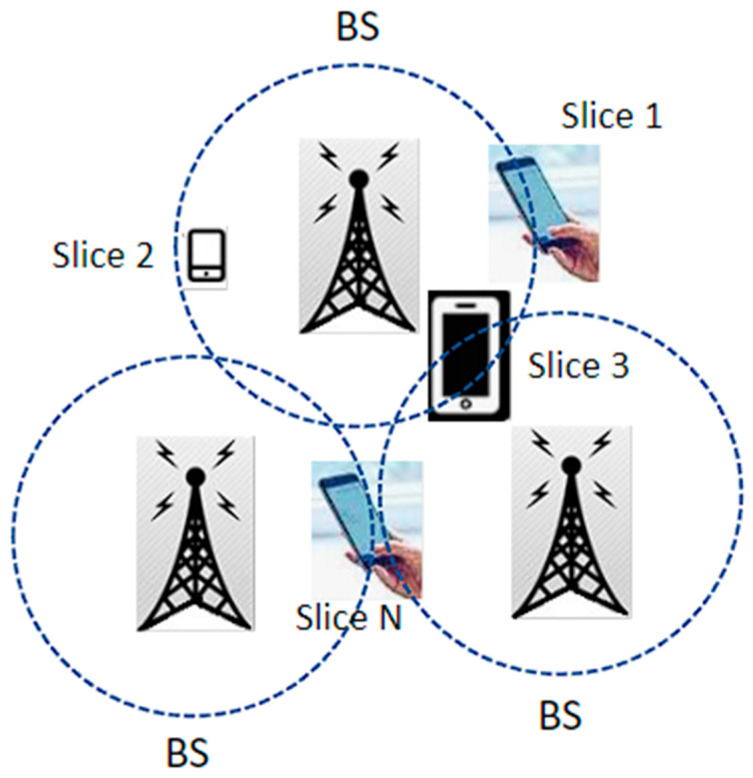
Network slicing illustration.

Network slicing (NS) represents the aggregation of any network flow as the traffic of operators (tenants, users, and infrastructure) that can be individually identified, controlled, and isolated. A network slice is a collection of mobile network functions and a set of Radio Access Technologies (RATs). These network functions combine the data class and pieces to adapt to the requirements of diverse applications. SDN and NFV are the techniques used for implementing NS. SDN is capable of centrally managing the network traffic for the software application. The role of NFV is to package network functions such as load balancing, firewalls, and routing that may be performed on commodity hardware devices [26]. Figure 6 illustrates a network architecture that supports network slicing to serve diverse customer needs.

The paper [27] reviews various aspects of 5G network slicing to enhance energy efficiency. It analyzes different 5G network slice use cases and defines KPIs for energy efficiency. The study proposes new techniques and algorithms to reduce energy consumption, including limiting slice energy use, dynamic state changes based on traffic variations, and resource adaptation in the time, frequency, and space domains. It also explores AI-based optimization processes and the spatial arrangement of baseband units in cloud data centers. Each technique analyzed contributes to improving 5G network energy efficiency, highlighting significant research potential to optimize energy consumption and reduce the environmental impact of energy-demanding 5G networks [27].

#### 3.2.1. Software-Defined Networking (SDN)

SDN separates the network control plane from the data plane, allowing for the centralized control and programmability of network resources. By dynamically managing and optimizing network traffic flows, SDN improves network efficiency, reduces power consumption, and enables more efficient resource allocation. In an SDN-based 5G network, the control plane, which is responsible for making decisions about traffic routing and resource allocation, is decoupled from the data plane, which handles the actual forwarding of data packets. 

**Figure 7:**
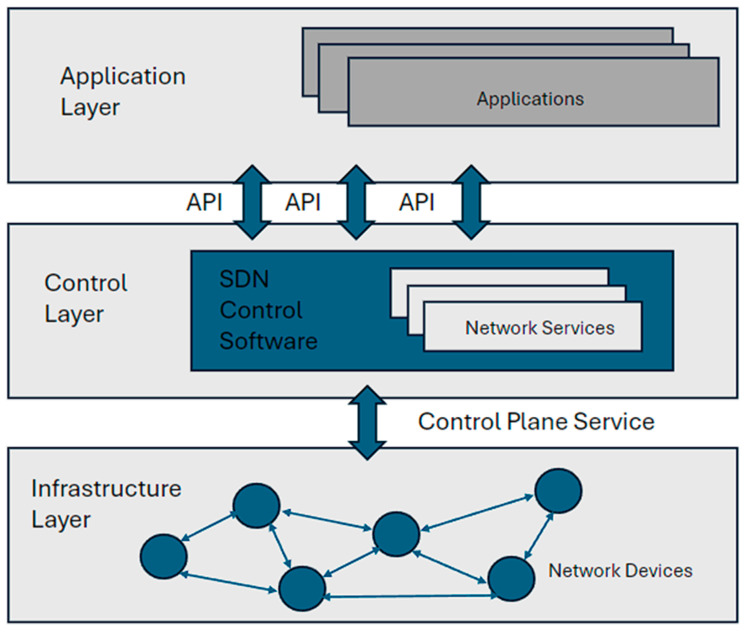
SDN architecture.

The control plane is centralized in a network controller, while the data plane consists of switches, routers, and other network devices. SDN enables the creation of network slices, which are virtualized, and independent end-to-end network instances tailored for specific use cases or services. Each network slice can have its network characteristics, such as bandwidth, latency, and security policies. SDN facilitates the dynamic provisioning, isolation, and management of network slices, allowing operators to efficiently serve diverse service requirements within a single physical infrastructure, as shown in Figure 7.

Hybrid Machine Learning Framework for Energy Efficient Routing in SDN (HyMER), a new hybrid ML framework powered by SDN and ML, allows traffic-aware, and energy-efficient routing at the core level [28]. The study conducted using HyMER heuristics showed that it can save up to 50% on link costs and consumes as little as 14.7 watts less for realistic traffic traces and network topologies. When compared to techniques that prioritize energy savings, it had a 2-hop shorter average path length, 15 Mbps higher throughput, and 5 ms shorter latency. HyMER maintained the trade-off between performance and energy efficiency, according to extensive tests. The HyMER delay was twenty percent to ninety percent for the Abilene and GEANT network topology and traffic traces. The results for throughput are like heuristics such as Next Shortest Path (NSP), Shortest Path First (SPF), and Next Maximum Utility (NMU), which prioritize performance while saving energy, achieving a delay of 9 ms for a 20% traffic volume and 11 ms for a 90% traffic volume [28]. 

A comparison of Highest Demand First (HDF) and Smallest Demand First (SDF) heuristics reveals that both are better for energy savings but suffer from a delay of 3 ms to 10 ms compared to NSP, NMU, and SPF. However, HyMER demonstrates a delay between the two types of heuristics [29]. In this experiment, the average bandwidth is 100 Mbps, and the average flow rates are 11.36 Mbps and 7.79 Mbps, respectively, for Abilene and Gigabit European Academic Network topologies and traffic traces [29].

In contrast, virtual machine-energy-saving approaches focus on minimizing the number of physical servers and management time involved with migration. The HyMER reinforcement component is the first to model both network performance and energy efficiency. They focused on the use of the HyMER algorithm for the delay metric to maximize QoS in terms of delay, congestion, and reliability, as well as to enhance security by detecting attacks in advance and classifying traffic capacity areas.

Another novel intelligent routing system for SDN is based on neural networks, whose data-flow transmission patterns are determined by using neural networks (NNs) instead of flow tables and by redeploying the NN packets with well-trained NNs [30]. By minimizing delivery times for packets, routing schemes are intended to reduce energy factors [3]. Therefore, energy efficiency and routing schemes are interrelated. A 20 MHz bandwidth and a total receiver noise power σ2 of −94 dBm are used to simulate communication with a maximum transmit power of 20 dBm per UE. Still, dropped calls, latency, throughput, peak data rate with coverage, and mobility were not emphasized. DNN is utilized to detect network traffic in the demo described in article [30].

#### 3.2.2. Network Function Virtualization (NFV)

NFV is an architectural approach in 5G networks that aims to virtualize and consolidate network functions onto standard hardware, such as servers, switches, and storage devices. NFV replaces traditional dedicated network equipment with software-based Virtualized Network Functions (VNFs) running on Virtual Machines (VMs) or containers. NFV enables the dynamic scaling of VNFs based on demand. By automatically scaling the number of active VNF instances according to real-time traffic load, operators can allocate resources only when necessary, reducing energy waste during periods of low traffic. This elastic scaling helps maintain energy efficiency by matching resource allocation to current needs. It allows for the consolidation of multiple network functions onto a shared hardware platform. By running multiple VNFs on the same physical server or data center, operators can achieve better resource utilization and reduce the number of active physical devices, leading to energy savings. VNF consolidation reduces the overall power consumption and footprint of the network infrastructure. 

**Figure 8:**
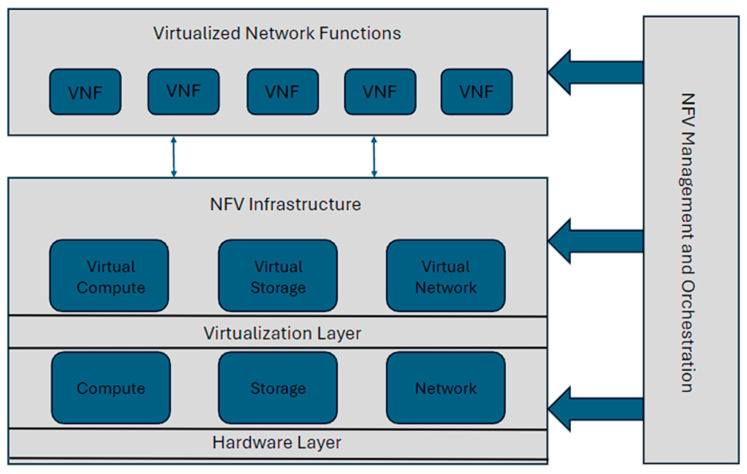
NFV architecture.

Higher energy efficiency is accomplished with resource allocation using a combination of NFV and LSTM compared to simple LSTM [31]. This model was created to achieve great accuracy in the forecasting of VNF resources. Instead of using simulations, an OpenStack-based test environment was used to demonstrate that this approach outperforms the standard model. Optimizing resource allocation for the related VNFs is one of the most critical concerns when evaluating service quality such as Service Function Chaining (SFC), which is necessary to avoid service interruptions owing to a shortage of resources during highly fluctuating traffic situations and to lower network operation costs. Figure 8 describes the architecture of NFV.

A resource allocation technique in NFV was proposed using DL [32]. It identifies network traffic by utilizing timing characteristics. The intelligent VNF was installed on a separate server with a Graphics Processing Unit (GPU) and operated on the data plane of SDN. The Mininet was used to construct the test bed and the environment’s architecture. The available connection bandwidth between each linked switch was set to 5 Mbps to 60 Mbps, and the link bandwidth between the switch and the host was set to 100 Mbs [32]. The delay between each connected switch was set to between 2 and 30 ms. To keep things simple, the authors chose four distinct types of apps with varying network requirements: Class 1 (real-time traffic), Class 2 (audio/video streaming), Class 3 (http browsing), and Class 4 (restricted/file transfers). The system could allocate various network resources to different applications, significantly improving the network QoS. VNF was added to the system to facilitate traffic identification to lessen the impact on the SDN controller. The network functions were therefore separated, allowing each module to operate independently. When the SDN controller conducted traffic identification, the introduction of VNF also solved the issue of high load [32].

### 3.3. Cloud and Edge Computing

In 5G networks, several cloud and edge technologies are utilized to enable efficient and scalable deployment. These technologies enhance the capabilities of 5G networks by providing computational resources, storage, and services closer to the network edge. Here are some key 5G cloud and edge technologies used in 5G networks:

#### 3.3.1. Cloud Radio Access Network (CRAN)

**Figure 9:**
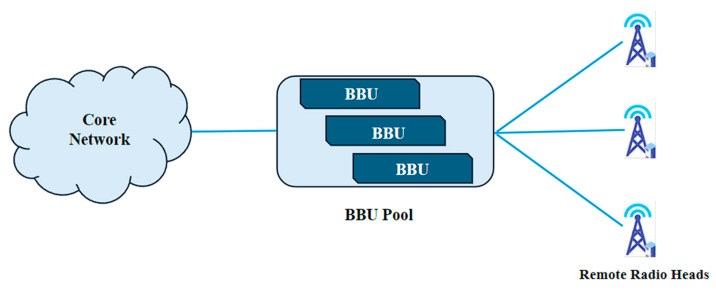
CRAN architecture.

CRAN is an architectural concept used in 5G networks that aims to centralize and virtualize the baseband processing of multiple BSs. In CRAN, the baseband processing functions, including signal processing, modulation/demodulation, coding/decoding, and resource allocation, are centralized in a central data center or cloud infrastructure. This centralization allows for the efficient pooling of baseband resources and enables more flexible and dynamic management of the radio access network. The Baseband Processing Units (BBUs) of multiple BSs are pooled together in the central data center, as shown in Figure 9. By sharing BBUs among multiple BSs, CRAN reduces the overall hardware and energy costs, as well as the complexity associated with maintaining and upgrading individual BBUs at each BS. 

Heterogeneous CRAN (H-CRAN) is a novel idea that facilitates the increase in capability for large-scale data rates. It enhances the QoS and improves energy efficiency. H-CRAN helps to reduce radio site operations costs, capital costs, and energy problems due to its architecture based on lower BS deployment. It is based on the use of high-power nodes (macro-BS and micro-BS) and low-power nodes (picocells and femtocells) to gather data. However, the use of low-power nodes will cause interference that affects energy consumption. Thus, controlling interference allows for a reduction in spectral efficiency deterioration and energy consumption. Furthermore, all of the following research in CRAN focuses on resource allocation, power minimization, and computational complexity using ML techniques. 

To improve the CRAN in the network, AI approaches are viewed as an optimization tool. In cloud computing platforms and to reduce power consumption, the sleeping cell idea is used with DNN [33]. Several cells can indeed be put into sleep mode to conserve the most power. Moreover, to tackle the problem in real-time applications, more time-efficient techniques are necessary [3]. ML is extremely important for separating distributed units and high-power nodes to reduce computing time and power consumption [3]. CRAN can efficiently process resources between cells and eliminate interference. This design change, however, introduces additional technological obstacles for execution. Furthermore, the proper deployment of wireless resources is still required to achieve improved power efficiency.

#### 3.3.2. Multi-Access Edge Computing (MEC)

**Figure 10:**
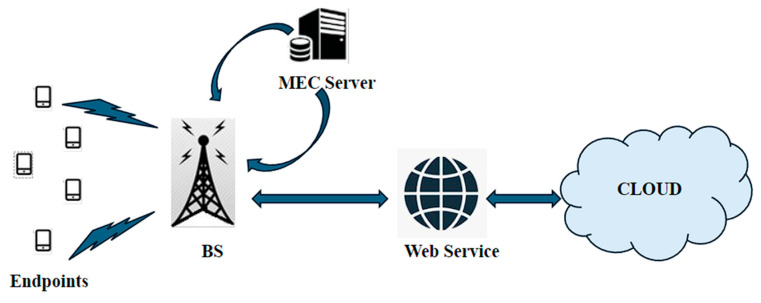
MEC architecture.

MEC is typically used in the access network layer of the 5G network architecture. MEC nodes are deployed at the edge of the network, usually at BSs or cell towers, to provide computing and storage resources closer to the end users and devices, as shown in Figure 10. MEC nodes are connected to the 5G core network, which enables seamless communication between the MEC nodes and other network functions such as the User Plane Function (UPF) and the Session Management Function (SMF). 

MEC is designed to support low-latency and high-bandwidth applications by enabling computation and data storage at the edge of the network. By moving computing and storage resources closer to the end users and devices, it can reduce the amount of data that need to be transmitted over the network, thereby reducing latency and improving overall network performance. It utilizes edge servers deployed at BSs or nearby data centers to provide computing resources at the network edge. The server can include processing power, storage, and networking capabilities. 

The infrastructure is designed to handle the specific requirements of mobile networks, enabling faster processing and reduced data transmission. MEC introduces a distributed architecture where computing resources are deployed at multiple points in the network. This allows for localized data processing and the offloading of certain tasks from centralized cloud servers, resulting in optimized network traffic and improved performance. 

MEC enables the development of innovative services and applications that benefit from the low latency and high bandwidth of 5G networks. Examples include augmented reality (AR) and virtual reality (VR) applications, real-time video analytics, autonomous vehicles, smart cities, and IoT deployments. MEC provides the necessary computing capabilities to support these latency-sensitive and bandwidth-intensive applications. It helps offload certain processing tasks from mobile devices to edge servers. This reduces the amount of data that need to be transmitted over the network, minimizing congestion and improving overall network efficiency. By processing data locally, MEC also reduces reliance on centralized cloud infrastructure, which can result in cost savings for data transfer and storage.

As for high-dimensional MEC situations, deep reinforcement learning (DRL) is an effective method for controlling complications [34]. A recurrent offloading choice was made using a computational technique, and the goal of this study was to reduce the total cost of all users in the MEC system by stressing latency performance and power consumption [35]. 

The authors considered a single small cell with a bandwidth of W = 10 MHz and an eNB installed with an MEC server in the center. The UEs were randomly distributed within a 200 m radius of the eNB. The MEC server’s processing capacity was F = 5 GHz/s, while the CPU frequency of each UE was 1 GHz/s. The transmission and idle power of the UE were set, in turn, to 500 mW and 100 mW [34]. Because there was just one eNB, they assumed that interval interference was ignored, and radio resources were not considered for this framework. However, they did not explore dropped calls or other important KPIs such as throughput, peak data rate, spectral data efficiency, user experience, UE battery life, and mobility.

### 3.4. Energy Harvesting

Fifth-generation energy harvesting refers to the process of capturing and utilizing ambient energy from the environment to power or supplement the energy needs of fifth-generation network devices and infrastructure. It involves converting various forms of energy, such as solar, wind, thermal, vibration, or radio frequency energy, into electrical energy that can be used to power 5G network components. Energy harvesting in 5G networks is particularly relevant due to the massive number of connected devices and the increasing demand for network coverage and capacity.

The demand for constant computing power for AI processing and the increasing popularity of IoT devices pose significant challenges to the energy efficiency of communication devices. Energy harvesting allows devices to power themselves, which is essential for off-grid operation, sustainable IoT devices and sensors, infrequently used devices, and long standby intervals. In addition, symbiotic wireless technology and smart power management technology offer potential solutions [36].

Wireless sensor networks (WSNs) consist of spatially distributed, inexpensive sensors for monitoring physical conditions. However, a major challenge for WSNs is their limited battery power. Energy harvesting, which involves converting ambient energy into electrical energy, is an effective alternative for powering WSNs, offering a sustainable solution to extend their operational lifetime and enhance their reliability.

Adapting Medium Access Control (MAC) protocols addresses specific aspects of energy harvesting by managing the active and sleep times of nodes based on the energy they have harvested [37]. Energy-aware MAC adapts node active times based on harvested energy levels, achieving high energy efficiency and fairness; radio frequency MAC prioritizes energy requests over data packets, leveraging constructive interference for higher throughput; the IEEE 802.15.4-based MAC for LTE RF energy harvesting optimizes energy usage through centralized control, while Harvest-then-Transmit-based MAC enhances energy harvesting rates and compatibility with WiFi standards, and the Dedicated RF Transmitter approach uses TDMA for energy and data transmission optimization, addressing fairness and efficiency challenges. These protocols collectively contribute to improving energy efficiency, data throughput, and network lifetime in RF energy harvesting systems. 

The proposed RF energy harvesting method in [38] enhances energy efficiency by optimizing the design of rectenna systems. By carefully selecting components such as antennas, impedance matching networks, and rectifiers, the system maximizes power conversion efficiency (PCE) and effectively utilizes both dedicated and ambient RF energy. Integrating reconfigurable rectifiers and boost converters further improves system performance. Additionally, the potential integration of hybrid energy harvesting, combining RF with other energy sources, offers a promising avenue for further enhancing energy efficiency. 

Another proposed method involves the comprehensive integration and optimization of various contemporary energy harvesting approaches tailored for WSNs [39]. This method utilizes multiple energy harvesting techniques, including vibrational (electromagnetic, electrostatic, piezoelectric), thermal (thermoelectric, pyroelectric), solar (photovoltaic), flow-based (hydrodynamic, aerodynamic), magnetic, and RF harvesting, ensuring the exploitation of the most abundantly available energy sources in the given environment. Efficient energy storage technologies, such as rechargeable batteries and supercapacitors, are incorporated based on their specific characteristics to ensure optimal energy retention and delivery. 

This method also implements different energy harvesting system topologies, including autonomous, autonomous-hybrid, and battery-supplemented models, to effectively combine energy harvesting and energy storage in powering the load. By maximizing power conversion efficiency and adapting the system to specific applications and environmental conditions, this approach enhances energy efficiency, reduces dependency on external power sources, and increases the operational lifetime and sustainability of WSN devices. 

#### 3.4.1. Millimeter Waves 

Millimeter wave (mmWave) is a spectrum frequency band that ranges from approximately 30 GHz to 300 GHz, since the Ka-band (18 GHz to 32 GHz) is also considered a mmWave [40]. This spectrum is suitable for high-speed wireless broadband communications because it is positioned between microwaves and infrared waves. It is used in the most recent 802.11 and Wi-Fi standards (functioning at 60 GHz) [41]. Due to the problem of bandwidth limitation and increased data demand in 5G networks, mmWave has been explored as a method to increase network capacity.

mmWave frequencies provide larger bandwidths, enabling significantly higher data transmission rates compared to lower frequency bands. This increased capacity allows for faster data transfer and supports the growing demand for high-bandwidth applications, such as streaming, virtual reality, and augmented reality. While mmWave technology offers higher capacity, it typically requires denser network deployments due to its shorter range and susceptibility to signal attenuation.

Denser networks can potentially lead to higher energy consumption as more BSs or small cells are required. Energy-efficient design and optimization strategies, such as intelligent resource allocation, sleep modes, and dynamic power management, are important for mitigating the increased energy demands of mmWave deployments. The rapidly expanding mobile industry, as well as the growing need for high data speeds, have prompted networks to open a new mmWave spectrum that can concurrently service a group of users utilizing beamforming [42].

Beamforming in mmWave technology is a critical technique used in 5G networks to overcome the challenges associated with high-frequency mmWave signals that enable efficient and reliable communication. It is a technique that uses multiple antennas to form a focused and directional signal beam. Instead of broadcasting signals uniformly in all directions, beamforming concentrates the transmitted energy in specific directions, increasing the signal strength and mitigating the effects of path loss and blockages. Beamforming is used in both the transmitter (transmit beamforming) and the receiver (receive beamforming). It utilizes advanced spatial signal processing algorithms to dynamically adjust the phase and amplitude of the signals at each antenna element. 

In practical implementation, hybrid beamforming is commonly used in mmWave 5G networks. Hybrid beamforming combines digital and analog beamforming techniques. Digital beamforming is performed at the baseband using digital signal processing, while analog beamforming is applied at the radio frequency level using phase shifters or analog components. This hybrid approach provides a trade-off between performance and complexity. Beamforming in mmWave 5G networks offers several advantages. It enables a longer range and better coverage by focusing the transmitted energy in the desired direction, which enhances energy efficiency and compensates for the high path loss of mmWave signals. 

**Figure 11:**
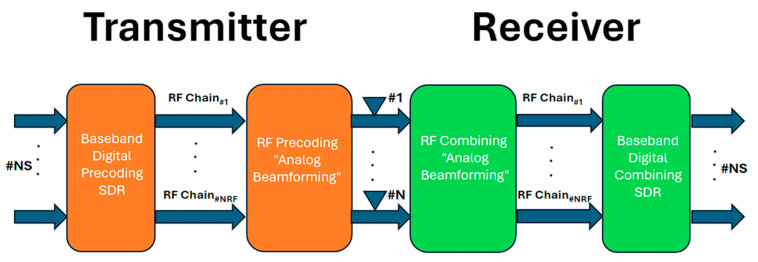
Hybrid beamforming architecture.

Beamforming also improves signal quality, enabling higher data rates and reducing interference from other devices and reflections. Therefore, beamforming has been embraced as a strategic approach for resolving interference between neighboring cells [43]. Figure 11 describes the architecture of hybrid beamforming.

Area communications using mmWaves suffer from significant propagation loss, high levels of attenuation, and diffraction [44]. The BSs that use mmWave are typically outfitted with massive arrays of antennas that aid in overcoming route loss, improving spectral efficiency, and increasing capacity. Energy efficiency is a challenge because of these enormous arrays of antennas. Deep learning-based methods can be performed by massively concurrent architectures with distributed memory architectures, such as GPUs. They have attracted attention because their energy efficiency and high throughput computing performance have piqued the curiosity of the industry.

#### 3.4.2. Heterogeneous Network (HetNet)

HetNets are characterized by the integration of diverse types of network nodes, such as macro cells, small cells, and Wi-Fi access points, within the same network. They are essential in 5G networks to address coverage gaps, increase network capacity, and provide seamless connectivity. 

**Figure 12:**
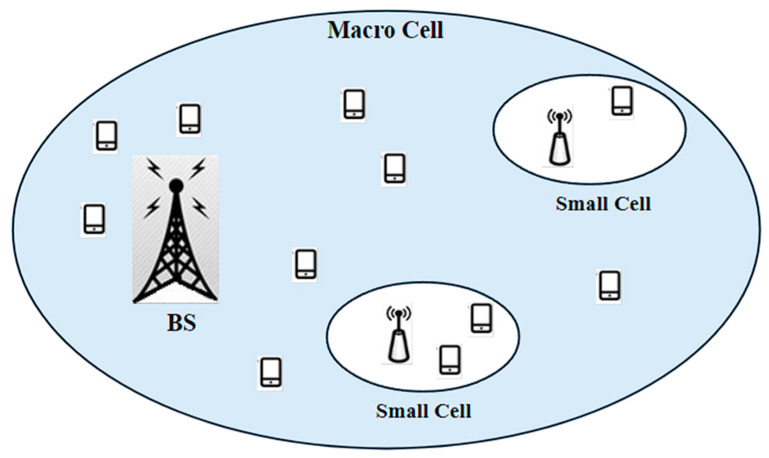
Heterogeneous network illustration.

They utilize a mix of different-sized cells, strategically deployed to optimize coverage and capacity in various areas. By deploying small cells or Wi-Fi access points in dense urban or high-traffic areas, the coverage and capacity can be enhanced while reducing the reliance on macro cells. This optimized coverage helps reduce energy consumption by avoiding the unnecessary transmission of signals over long distances. The deployment of different kinds of cells is shown in Figure 12.

In [45] the energy efficiency of cellular networks, particularly those with macro-BSSs and new technologies like LTE, were investigated. The focus was on two key metrics: Power per Unit Area and Energy per bit and Unit Area. Analytical models were developed to show how the ways in which base stations transmit power, inter-site distances, and the presence of macro- and micro-LTE BSSs affect these metrics. The study demonstrates that the optimal selection of these parameters can lead to significant energy savings. Adding micro-LTE BSs to heterogeneous networks further enhances energy efficiency. The simulation results of various network configurations highlight potential energy savings for network operators, reflecting scenarios similar to current or anticipated cellular networks [45]. 

HetNets enable the offloading of traffic from macro cells to small cells or Wi-Fi networks, relieving congestion and increasing overall network capacity. By utilizing small cells or Wi-Fi access points where appropriate, HetNets can better distribute network load, reducing the energy consumption associated with handling high traffic volumes in macro cells. They can benefit from energy harvesting technologies such as solar panels or radio frequency energy harvesting. These techniques can provide a supplemental power source, reducing the reliance on electrical grid power and enabling energy-autonomous operation for small cell deployments.

When planning and optimizing HetNet deployments, energy harvesting potential should be considered. Assessing ambient energy sources like solar or radio frequency signals and incorporating energy harvesting capabilities into the network infrastructure can enhance energy efficiency and reduce the network’s carbon footprint.

DRL is used to tackle energy efficiency in uplink HetNets as well as resource allocation optimization [3]. But the spectrum reuse causes interference between heterogeneous cell sizes (like pico, femto, macro, small). Small cells can assist the network in meeting the data demands of several connected devices as well as large data traffic with high communication data rates. They are vulnerable to significant power consumption [3]. 

In a simulation, co-channel interference increases with the growing number of UEs [46]. When pico- and femto-BSs overlap with macro-BS coverage, this interference becomes apparent. Reference [47] examines the impact of randomly distributed UEs within macro-BS coverage. HetNets demonstrate potential for enhancing spectrum efficiency by allowing pico- and femto-BSs to share channels with macro-BSs, thereby boosting system capacity for future mobile communications. In summary, the simulation highlights the challenges posed by co-channel interference in mixed macro-, pico-, and femto-BS deployments, emphasizing the role of HetNets in optimizing system capacity for upcoming mobile communication needs [46,47]. The Dueling Double Deep Q-Network technique proposed for addressing large-scale learning challenges offers advantages in improving system capacity and network utility with reduced processing time. There are limitations in the absence of dropped calls, throughput, latency, UE battery life, and mobility KPIs.

### 3.5. KPIs for Different ML Techniques

Several KPIs have been studied for different ML techniques used in each 5G enabler. Table 2 presents a comparison of ML techniques in 5G green enablers based on the specified KPIs.

Massive MIMO, employing a max–min approach and neural networks in LSTM layers, focuses on enhancing area traffic capacity and extending battery life without specifying throughput or latency improvements. O-RAN utilizes reinforcement learning with Q-learning and SARSA algorithms to improve latency, energy efficiency, and QoS, making strides in spectral data efficiency while not directly addressing throughput or system bandwidth. 

NOMA, through iterative algorithms based on CGP, enhances energy efficiency and coverage but lacks specific metrics on latency or QoS. TDMA, using the Lagrange dual method, optimizes throughput and energy efficiency but does not impact latency or system bandwidth directly. PD-NOMA employs heuristic joint subcarrier and power allocation schemes to enhance latency, peak data rate, and battery life, focusing on comprehensive coverage and the efficient use of resources. 

SDN, leveraging hybrid machine learning approaches and APC-III with K-means, enhances QoS and area traffic capacity while facilitating dynamic network management and resource allocation, demonstrating versatility in improving 5G network performance. 

NFV combined with LSTM aims to leverage memory-based learning for network function virtualization, yet specific metrics like throughput, latency, and energy efficiency are not addressed. On the other hand, NFV integrated with deep learning shows promise in enhancing system performance with improvements in latency, QoS, and battery life. 

CRAN utilizes deep neural networks to optimize throughput and energy efficiency while supporting high QoS standards, but lacks specifics on latency and system bandwidth improvements. MEC employs computational offloading solutions to enhance latency and area traffic capacity, focusing on dynamic decision-making processes. 

HetNets leverage deep reinforcement learning to improve overall network efficiency and coverage, particularly in managing heterogeneous network environments. Lastly, mmWave technologies utilize deep learning for precoding to enhance energy efficiency, targeting advancements in throughput and spectral data efficiency without directly addressing latency or QoS metrics.

## 4. Future Directions

Energy efficiency is a critical aspect of 5G networks, and further research is needed to optimize energy consumption and improve sustainability. One potential avenue for exploration is the integration of CGP in SON to enhance energy efficiency. The following areas can be addressed in future research:

- Energy-efficient network design: Further research can focus on developing energy-efficient network architectures and protocols specifically designed for 5G networks. Investigating the trade-offs between energy consumption and network performance metrics, such as latency, throughput, and reliability, can help optimize network design. By leveraging CGP algorithms, network architectures can be optimized to reduce energy consumption while maintaining satisfactory performance levels.
- Power management techniques: Exploring advanced power management techniques can significantly contribute to reducing energy consumption in 5G networks. CGP can be used to develop intelligent algorithms that dynamically adjust the power levels or optimize the operational parameters of network elements. This can include adaptive power control, dynamic sleep modes, or efficient resource allocation, ensuring energy is utilized optimally without compromising network performance.
- Energy harvesting integration: Integrating energy harvesting techniques in 5G networks can contribute to reducing energy consumption and promoting sustainability. CGP algorithms can be used to optimize the integration of energy harvesting sources, such as solar panels or RF energy harvesting, into network components. This allows for the efficient utilization of harvested energy to power network elements and reduces reliance on conventional energy sources.
- Energy-aware self-organizing algorithms: SONs can benefit from CGP by developing energy-aware self-organizing algorithms. These algorithms can analyze real time energy consumption data and network conditions to dynamically optimize network parameters, such as coverage, handover algorithms, or resource allocation, while considering energy efficiency as a key objective. CGP-based algorithms can adaptively adjust network configurations to minimize energy consumption while maintaining the desired network performance.
- Dynamic network optimization: CGP algorithms can be utilized to enable dynamic network optimization in SON environments. By continuously analyzing network data and performance metrics, CGP-based algorithms can dynamically adjust network parameters to achieve optimal energy efficiency. This adaptive optimization approach ensures that the network operates efficiently under varying conditions and traffic patterns.

To validate the effectiveness of the proposed CGP technique for dynamic network optimization in SON environments, consider a 5G cellular network scenario. Here, CGP algorithms would be trained using historical network data to recognize patterns and optimize parameters. In real time, the CGP algorithms would dynamically adjust transmit power levels, antenna configurations, and channel assignments based on real-time data from network nodes to optimize performance and energy efficiency. By continuously analyzing network conditions and user demands, the CGP-based system would reduce energy consumption, improve throughput and latency, and enhance signal quality. Simulations would demonstrate the system’s ability to adapt to varying conditions, showcasing its practical benefits and robustness.

The CGP algorithm can address dynamic network optimization challenges posed by changing channel conditions through adjustments in its fitness function and the incorporation of adaptive mechanisms. In large-scale network models, reducing energy consumption during CGP training can be achieved by optimizing data handling, leveraging parallel computing, enhancing algorithmic efficiency, and developing energy-efficient variants of CGP. In addition to conventional performance metrics such as accuracy and throughput, it is advisable to consider metrics that assess energy efficiency, robustness, scalability, adaptability, and convergence speed to comprehensively evaluate the effectiveness of the optimized network solutions. 

The integration of edge computing and MEC is another promising area. Advancing research on edge computing architectures and frameworks to support low-latency and high-bandwidth applications in 5G networks will be crucial. Additionally, exploring the integration of AI and ML algorithms at the edge to enable real-time data processing, enhance energy efficiency, and improve decision-making is essential. Developing energy-efficient communication protocols and algorithms tailored for 5G networks is important. These protocols should consider the unique characteristics of diverse use cases and traffic patterns. Investigating energy-saving mechanisms, such as adaptive modulation and coding, sleep mode operation, and traffic offloading strategies, will contribute to reducing energy consumption in wireless communications. Additionally, exploring cross-layer optimization techniques that consider both physical layer considerations and higher-layer network protocols will contribute to improved energy efficiency. 

Optimizing network slicing techniques is another important direction for future research. This involves exploring innovative approaches to dynamically allocating network slices based on specific application requirements, such as latency, bandwidth, and reliability. Further investigation is needed to understand the impact of network slicing on network performance, scalability, and cost-effectiveness and to identify ways to optimize slice management and orchestration.

Energy efficiency is a key aspect of 5G networks, and further research is needed to develop energy-efficient network architectures and protocols specifically designed for 5G. Investigating the trade-offs between energy consumption and network performance metrics to optimize network design is important. Advanced power management techniques, such as dynamic sleep modes, energy harvesting, and intelligent resource allocation, can be explored to reduce energy consumption in 5G networks. By addressing the mentioned technologies, further research can be conducted on developing more advanced AI algorithms and models to enhance natural language processing, computer vision, and decision-making capabilities. This will enable more intelligent and context-aware network operations.

## 5. Conclusions

In conclusion, this research paper has provided an in-depth analysis of the 5G network, focusing on its various layers and enablers, and the application of AI-based techniques to improve energy efficiency and overall performance. Throughout this paper, we have emphasized the importance of energy efficiency in implementing environmentally friendly strategies within the 5G network. By examining the different layers of the network, we have identified key enablers and their characteristics, shedding light on the underlying technologies that drive the 5G network’s functionality. Additionally, we have explored how AI techniques can be employed to enhance energy efficiency and optimize KPIs, contributing to a more sustainable and efficient network infrastructure. Furthermore, this paper has outlined potential directions and challenges for future research in the field of 5G networks. By identifying areas that require further investigation and addressing potential obstacles, we hope to inspire and guide scholars in their continued exploration of this rapidly evolving domain. In summary, this research paper outlines the significance of energy efficiency in sustainable 5G networks. Through the application of AI and addressing challenges, it promises improved performance and reduced energy consumption for a greener telecommunications approach.

## Figures and Tables

**Table 1:** ML techniques in 5G enablers.

Network Level	5G Enabler	ML Technique
RAN	Massive MIMO	Max–min and maximum production approaches shows incompetence, which is then addressed through a different neural network using the LSTM layer. DL techniques max–min and max-prod power allocation are used in the downlink of massive MIMO networks to learn the map between the positions of UEs, the optimal power allocation, and the forecast power allocation profiles for new UE placements. This technique improves power allocation compared to traditional optimization methods. In addition, the spectrum efficiency of hybrid precoding reduces the RF chains’ huge energy consumption in the massive MIMO system
	NOMA	An iterative algorithm based on CGP was implemented. BS uses NOMA for downlink transmission to UEs. The resource allocation issue, which seeks to reduce total transmit power while considering isolation limitations, is non-convex and has a high computing cost. The suggested method surpasses O-FDMA in terms of necessary transmit power, especially when most users are situated in the same area. As a result, it is worth looking into the power efficiency of NOMA in a multi-cell situation.
	PD-NOMA	A sensor field of K IoT sensors, BNs, and a reader is considered to adopt a monostatic backscatter communication model to improve network energy efficiency. An efficient heuristic JSPA is designed by combining the solution of SCUS and MCPC.
	TDMA	Adopting the Lagrange dual method of joint time and power allocation in RAN showed that TDMA beat NOMA, demonstrating that TDMA is more spectral and energy-efficient. NOMA requires longer (or equal) downlink time than TDMA, consumes more, or equal, energy, and has lower spectral efficiency.
	O-RAN	An RLDFS technique that decides on the function splits in an O-RAN is implemented to make the best use of renewable energy supply and minimize operator costs by using Q-Learning and SARSA algorithms. RLDFS is applied to an actual data set of solar irradiation and traffic rate fluctuations to evaluate the performance of the suggested technique. MNO should choose the right size of solar panels and battery capacity to save renewable energy.
Core network	SDN	HyMER algorithms have demonstrated up to 50% link savings and up to 14.7 watts of reduced power usage for realistic network topology and traffic traces. The HyMER framework is built on the POX controller and tested on Mininet with real-world topologies and dynamic traffic traces. Hymer maintains the trade-off between network performance and energy efficiency. Access Point Controller (APC) K-means combines APC-III with K-means, which allows the calculation of radial basis function centers. The simulation results further demonstrate the feasibility and effectiveness of the proposed neural network-based Intelligent Routing Strategy for SDN (NNIRSS).
	NFV	Energy efficiency is accomplished with resource allocation using a combination of NFV and LSTM compared to simple LSTM. This model was created to achieve great accuracy in the forecasting of VNF resources. Instead of using simulations, an OpenStack-based test environment was used to demonstrate that this approach outperforms the standard model. Optimizing the resource allocation of the related VNFs is one of the most critical concerns when evaluating the service quality of such an SFC, which is necessary to avoid service interruptions owing to a shortage of resources during highly fluctuating traffic situations and to lower network operation costs.
Edge computing	CRAN	The cell sleeping concept is used to minimize power consumption along with DNN, incorporating sleep mode with associated transmission links and optimizing beamforming weights. Implementing this architectural shift presents new technical challenges as well. Allocating wireless resources efficiently is another challenge that must be met to achieve higher power efficiency.
	MEC	A computational offloading solution was employed to make the recurrent offloading decision. To make the final decision on recurrent offloading, a computational offloading solution was implemented on the CPU. In MEC, other factors, such as radio resources, predominate over computational requirements.
Energy harvesting	mmWaves	A beamforming scheme using DL for precoding enhances energy efficiency. It is implemented in BSs using mmWave with massive arrays of antennas.
	HetNets	DRL, used in macro-, pico-, and femto-BSs, can solve decision-making and resource allocation problems efficiently in real time. Energy consumption is solved in uplink HetNet along with user association optimization using DRL. Its disadvantage is that small and microcells interfere with each other due to spectrum reuse. The small cells can provide the network with the data to connect several devices and massive data traffic for communication with high data rates. However, they consume excessive amounts of energy.

**Table 2:** Comparison of KPIs for different ML techniques.

5G Enablers	ML Technique	Throughput	Latency	Energy Efficiency	Peak Data Rate	Spectral Data Efficiency	QoS	Area Traffic Capacity	System BW	Battery Life	Coverage
Massive MIMO	Max–min approach + Neural network in LSTM layer	NA	NA	NA	NA	NA	NA	NA	Yes	NA	Yes
O-RAN	A reinforcement learning + Q-learning and SARSA algorithm	NA	Yes	Yes	NA	NA	Yes	NA	NA	Yes	NA
NOMA	Iterative algorithm based on CGP	NA	NA	Yes	NA	Yes	NA	NA	NA	NA	Yes
TDMA	Lagrange dual method	Yes	NA	Yes	NA	Yes	NA	NA	NA	NA	Yes
PD-NOMA	A sensor field of K IoT sensors, BNs, and reader	Yes	NA	Yes	Yes	Yes	NA	Yes	NA	Yes	Yes
PD-NOMA	Heuristic joint subcarrier and power allocation scheme	NA	Yes	NA	NA	NA	Yes	NA	Yes	NA	Yes
SDN	Hybrid machine learning	Yes	Yes	Yes	NA	NA	Yes	NA	Yes	NA	NA
SDN	APC-III with K-means	NA	NA	NA	NA	NA	NA	Yes	Yes	NA	Yes
NFV	Combination of NFV and Long Short-Term Memory	NA	NA	NA	NA	NA	NA	NA	NA	NA	NA
NFV	Deep learning	NA	Yes	Yes	NA	NA	Yes	NA	NA	NA	Yes
CRAN	Deep neural network	NA	Yes	Yes	NA	NA	Yes	NA	NA	NA	Yes
MEC	Computational offloading solution for recurrent decision	NA	Yes	Yes	NA	NA	NA	NA	Yes	NA	Yes
HetNets	Deep reinforcement learning	NA	NA	NA	NA	Yes	Yes	Yes	Yes	NA	Yes
MmWave	Deep learning for precoding enhanced energy efficiency	NA	NA	Yes	NA	Yes	NA	NA	NA	NA	NA

## Data Availability

Data are contained within the article.

## Abbreviations

5G	Fifth generation
AI	Artificial intelligence
AR	Augmented reality
BBUs	Baseband Processing Units
BN	Backscatter Node
BSSs	Base Station Systems
CDMA	Code Division Multiple Access
CGP	Complementary Geometric Programming
CNN	Convolutional neural network
CU	Centralized unit
C-RAN	Centralized RAN
DL	Deep learning
DNN	Deep neural network
D-RAN	Distributed RAN
DRL	Deep reinforcement learning
DU	Distributed unit
DSC	Deadline Scheduling with Commitment
EDF	Earliest Deadline First
eMBB	Enhanced Mobile Broadband
FDMA	Frequency Division Multiple Access
GPU	Graphics Processing Unit
HDF	Highest Demand First
H-CRAN	Heterogeneous CRAN
HETNET	Heterogeneous network
HPA	Higher-Power Amplifiers
ICT	Information and Communication Technology
ILP	Integer Linear Programming
IoT	Internet of Things
JSPA	Joint subcarrier and power allocation
KPIs	Key Performance Indicators
LTE	Long-Term Evolution
LSTM	Long Short-Term Memory
MAC	Medium Access Control
MEC	Multi-access edge computing
MCPC	Multiple Channels Per Carrier
MIMO	Multiple Input Multiple Output
MNO	Mobile Network Operator
ML	Machine learning
MMWAVES	Millimeter Waves
NFV	Network function virtualization
NOMA	Non-Orthogonal Multiple Access
NNs	Neural networks
NS	Network slicing
NSP	Next Shortest Path
NMU	Next Maximum Utility
O-FDMA	Orthogonal FDMA
OMA	Orthogonal Multiple Access
O-RAN	Open radio access network
OFDM	Orthogonal Frequency Division Multiplexing
PD-NOMA	Power Domain Non-Orthogonal Multiple Access
PCE	Power conversion efficiency
QoS	Quality of service
RAN	Radio access network
RATs	Radio Access Technologies
RB	Resource block
RES	Renewable energy sources
RLDFS	Reinforcement learning dynamic function splitting
RF	Radio frequency
RNN	Recurrent neural networks
SFC	Service Function Chaining
SCUS	System Communication Units
SDN	Software-defined networking
SIC	Successive Interference Cancelation
SMF	Session Management Function
SPF	Shortest Path First
SDF	Smallest Demand First
TDMA	Time Division Multiple Access
UE	User Equipment
UPF	User Plane Function
URLLC	Ultra-Reliable Low Latency Communication
VMs	Virtual Machines
VNF	Virtualized Network Function
VR	Virtual reality
VRAN	Virtual radio access network
VWN	Virtualized Wireless Networks
WSNs	Wireless sensor networks
WSR	Weak State Routing

## References

[1] Bohli A., Bouallegue R. (2019). How to Meet Increased Capacities by Future Green 5G Networks: A Survey. IEEE Access.

[2] Huang H., Song Y., Yang J., Gui G., Adachi F. (2019). Deep-Learning-Based Millimeter-Wave Massive MIMO for Hybrid Precoding. IEEE Trans. Veh. Technol..

[3] Mughees A., Huang H., Song Y., Yang J., Gui G., Adachi F. (2020). Towards Energy Efficient 5G Networks Using Machine Learning: Taxonomy, Research Challenges, and Future Research Directions. IEEE Access.

[4] Nyalapelli A., Sharma S., Phadnis P., Patil M., Tandle A. Recent Advancements in Applications of Artificial Intelligence and Machine Learning for 5G Technology: A Review. Proceedings of the 2023 2nd International Conference on Paradigm Shifts in Communications Embedded Systems, Machine Learning and Signal Processing (PCEMS).

[5] Gunturu V., Reddy B., Sharma P., Singh A., Zhang L. Artificial Intelligence Integrated with 5G for Future Wireless Networks. Proceedings of the 2023 International Conference on Inventive Computation Technologies (ICICT).

[6] Larsen L.M.P., Johnson T., Smith R. (2023). Toward Greener 5G and Beyond Radio Access Networks—A Survey. IEEE Open J. Commun. Soc..

[7] Arjoune Y., Faruque S. Artificial Intelligence for 5G Wireless Systems: Opportunities, Challenges, and Future Research Direction. Proceedings of the 2020 10th Annual Computing and Communication Workshop and Conference (CCWC).

[8] Ghag O.M. (2023). Artificial Intelligence and Energy Efficiency of 5G Radio Access Network. Int. J. Comput. Eng..

[9] Lorincz J., Capone A., Begušić D. (2011). Heuristic Algorithms for Optimization of Energy Consumption in Wireless Access Networks. KSII Trans. Internet Inf..

[10] Lorincz J., Bogarelli M., Capone A., Begušić D. Heuristic Approach for Optimized Energy Savings in Wireless Access Networks. Proceedings of the 18th International Conference on Software, Telecommunications and Computer Networks (SoftCOM2010).

[11] Lorincz J., Bule I., Kapov M. (2014). Performance Analyses of Renewable and Fuel Power Supply Systems for Different Base Station Sites. Energies.

[12] Al-Khafaji M., Elwiya L. ML/AI Empowered 5G and Beyond Networks. Proceedings of the 2022 International Congress on Human-Computer Interaction, Optimization and Robotic Applications (HORA).

[13] Sanguinetti L., Zappone A., Debbah M. Deep Learning Power Allocation in Massive MIMO. Proceedings of the 2018 52nd Asilomar Conference on Signals, Systems, and Computers.

[14] Rommel S., Raddo R.T., Monroy I.T. The Fronthaul Infrastructure of 5G Mobile Networks. Proceedings of the IEEE 23rd International Workshop on Computer Aided Modeling and Design of Communication Links and Networks (CAMAD).

[15] Orumwense E.F., Abo-Al-Ez K. (2023). An Optimal Scheduling Technique for Smart Grid Communications over 5G Networks. Appl. Sci..

[16] Ismail S., D’andreagiovanni F., Lakhlef H., Imine Y. Recent Advances on 5G Resource Allocation Problem Using PD-NOMA. Proceedings of the 2020 International Symposium on Networks, Computers and Communications (ISNCC).

[17] Bai L., Zhu L., Yu Q., Choi J., Zhuang W. (2019). Transmit Power Minimization for Vector-Perturbation Based NOMA Systems: A Suboptimal Beamforming Approach. IEEE Trans. Wirel. Commun..

[18] Dawadi R., Parsaeefard S., Derakhshani M., Le-Ngoc T. Power-Efficient Resource Allocation in NOMA Virtualized Wireless Networks. Proceedings of the IEEE Global Communications Conference (GLOBECOM).

[19] Wu Q., Chen W., Ng D.W.K., Schober R. (2018). Spectral and Energy-Efficient Wireless Powered IoT Networks: NOMA or TDMA?. IEEE Trans. Veh. Technol..

[20] Zeb S., Abbas Q., Hassan S.A., Mahmood A., Mumtaz R., Zaidi S.M.H., Zaidi S.A.R., Gidlund M. NOMA Enhanced Backscatter Communication for Green IoT Networks. Proceedings of the International Symposium on Wireless Communication Systems (ISWCS).

[21] Salaün L., Coupechoux M., Chen C.S. Weighted Sum-Rate Maximization in Multi-Carrier NOMA with Cellular Power Constraint. Proceedings of the IEEE INFOCOM 2019—IEEE Conference on Computer Communications.

[22] Bai L., Zhu L., Zhang X., Zhang W., Yu Q. (2018). Multi Satellite Relay Transmission in 5G: Concepts, Techniques, and Challenges. IEEE Network.

[23] Gavrilovska L., Rakovic V., Denkovski D. (2020). From Cloud RAN to Open RAN. Wirel. Pers. Commun..

[24] Pamuklu T., Erol-Kantarci M., Ersoy C. Reinforcement Learning Based Dynamic Function Splitting in Disaggregated Green Open RANs. Proceedings of the IEEE International Conference on Communications.

[25] Srinivas K., Aswini J., Patro P., Kumar D. Functional Overview of Integration of AIML with 5G and Beyond the Network. Proceedings of the 2023 International Conference on Computer Communication and Informatics (ICCCI).

[26] Debbabi F., Rihab J.M.A.L., Chaari L., Aguiar R.L., Gnichi R., Taleb S. Overview of AI-Based Algorithms for Network Slicing Resource Management in B5G and 6G. Proceedings of the 2022 International Wireless Communications and Mobile Computing (IWCMC).

[27] Lorincz J., Kukuruzović A., Blažević Z. (2024). A Comprehensive Overview of Network Slicing for Improving the Energy Efficiency of Fifth-Generation Networks. Sensors.

[28] Assefa B.G., Ozkasap O. (2019). Hymer: A Hybrid Machine Learning Framework for Energy Efficient Routing in SDN. arXiv.

[29] Osseiran A., Monserrat J.F., Marsch P. (2016). 5G Mobile and Wireless Communications Technology.

[30] Uddin M., Amin M.R., Ahammad M.G. (2019). Nnirss: Neural Network-Based Intelligent Routing Scheme for SDN. Neural Comput. Appl..

[31] Kim H.-G., Lee D.Y., Jeong S.Y., Choi H., Yoo J.H., Hong J.W.K. Machine Learning-Based Method for Prediction of Virtual Network Function Resource Demands. Proceedings of the 2019 IEEE Conference on Network Softwarization (NetSoft).

[32] Xu J., Wang J., Qi Q., Sun H., He B. IARA: An Intelligent Application-Aware VNF for Network Resource Allocation with Deep Learning. Proceedings of the 2018 15th Annual IEEE International Conference on Sensing, Communication, and Networking (SECON).

[33] Du G., Wang L., Liao Q., Hu H. Deep Neural Network-Based Cell Sleeping Control and Beamforming Optimization in Cloud-RAN. Proceedings of the IEEE 90th Vehicular Technology Conference (VTC2019-Fall).

[34] Liaqat M., Noordin K.A., Abdul Latef T., Dimyati K. (2020). Power-Domain Non-Orthogonal Multiple Access (PD-NOMA) in Cooperative Networks: An Overview. Wirel. Networks.

[35] Li J., Gao H., Lv T., Lu Y. Deep Reinforcement Learning Based Computation Offloading and Resource Allocation for MEC. Proceedings of the IEEE Wireless Communications and Networking Conference (WCNC).

[36] Aneesh S., Shaikh A.N. A Survey for 6G Network: Requirements, Technologies and Research Areas. Proceedings of the 2023 2nd International Conference on Edge Computing and Applications (ICECAA).

[37] Sherazi H.H.R., Zorbas D., O’Flynn B. (2022). A Comprehensive Survey on RF Energy Harvesting: Applications and Performance Determinants. Sensors.

[38] Bougas I.D., Papadopoulou M.S., Boursianis A.D., Kokkinidis K., Goudos S.K. (2021). State-of-the-Art Techniques in RF Energy Harvesting Circuits. Telecom.

[39] Williams A.J., Torquato M.F., Cameron I.M., Fahmy A.A., Sienz J. (2021). Survey of Energy Harvesting Technologies for Wireless Sensor Networks. IEEE Access.

[40] Ahmad W.S.H.M.W., Radzi N.A.M., Samidi F.S., Ismail A., Abdullah F., Jamaludin M.Z., Zakaria M. (2020). 5G Technology: Towards Dynamic Spectrum Sharing Using Cognitive Radio Networks. IEEE Access.

[41] Baumgartner M., Juhar J., Papaj J. Simulation of 5G and LTE-A Access Technologies via Network Simulator NS-3. Proceedings of the 2021 44th International Conference on Telecommunications and Signal Processing (TSP).

[42] Chen J., Chen S., Qi Y., Fu S. (2019). Intelligent Massive MIMO Antenna Selection Using Monte Carlo Tree Search. IEEE Trans. Signal Process..

[43] El Hassani S., El Hassani H., Boutammachte N. (2019). Overview on 5G Radio Frequency Energy Harvesting. ASTESJ.

[44] Singh S.K., Singh R., Kumbhani B. The Evolution of Radio Access Network towards Open-RAN: Challenges and Opportunities. Proceedings of the 2020 IEEE Wireless Communications and Networking Conference Workshops (WCNCW).

[45] Lorincz J., Matijevic T. (2014). Energy-efficiency analyses of heterogeneous macro and micro base station sites. Comput. Electr. Eng..

[46] Zhao N., Liang Y.C., Niyato D., Pei Y., Wu M., Jiang Y. (2019). Deep Reinforcement Learning for User Association and Resource Allocation in Heterogeneous Cellular Networks. IEEE Trans. Wirel. Commun..

[47] Ding H., Zhao F., Tian J., Li D., Zhang H. (2020). A Deep Reinforcement Learning for User Association and Power Control in Heterogeneous Networks. Ad. Hoc. Netw..

